# Psychodynamic treatments taking an embodied perspective and their effect on depressive symptomatology: a systematic review and meta-analysis of randomized controlled trials

**DOI:** 10.3389/fpsyg.2026.1727473

**Published:** 2026-03-26

**Authors:** Maximilian Heider, Serge Sulz, Georg Franzen

**Affiliations:** 1 Department of Psychotherapy Science, Sigmund Freud Private University, Berlin, Germany; 2 Faculty of Philosophy and Education, Eichstätt KU, Eichstätt, Germany

**Keywords:** depression, embodiment, meta-analysis, psychodynamic psychotherapy, psychotherapy outcome

## Abstract

**Introduction:**

Within a psychodynamic framework multiple theoretical models are offered in the treatment of depression and some of these models also integrate the subjective experience of the body. We give a brief overview of the subjective body in psychodynamic psychotherapies and the evidence regarding the efficacy of psychodynamic treatments addressing the subjective bodily experience in depression. The subsequent review then aimed at identifying the interventions applied within psychodynamic treatments taking an embodied perspective in the treatment of depression while also aggregating findings on their average efficacy.

**Methods:**

A systematic review was carried out on RCTs comparing psychodynamic psychotherapy (PDP) addressing the subjective body to control conditions or bona fide therapies, the latter are therapies that have shown their efficacy in a broad context already. Outcomes were analyzed quantitatively using random effects models. The main characteristics of the applied treatment models were summarized narratively from the underlying intervention descriptions. Findings on the treatment models were structured in clusters according to their theoretical embedding.

**Results:**

Eleven Trials matched the inclusion criteria with a total of 1,624 patients. Post treatment PDP addressing the subjective body differed from control conditions with a standardized mean difference (SMD) = −0.53 [*p* = 0.017, 95% CI (−0.97, −0.09), *I*^2^ = 89.12%]. At follow-up differences to control conditions remained to be present with SMD = −1.23 [*p* = 0.005, 95% CI (−2.10, −0.35), *I*^2^ = 90.35%]. Compared to bona fide therapies no differences were detected post-treatment when applying two-one-sided-tests [SMD = 0.08, *p* = 0.039, 90% CI (−0.064, 0.23)]. Throughout the applied interventions three overarching technical principles were identified. Concurrently marked differences existed.

**Conclusion:**

The findings of our study suggest that PDPs addressing the subjective body are potentially efficacious. However, further conceptual elaboration on embodied treatment techniques is necessary. Subsequent studies should, in addition, scrutinize the contribution that these specific interventions make at the process and outcome level.

**Systematic review registration:**

PROSPERO, identifier (CRD42024523337).

## Introduction

Depression is characterized not only by mood alterations, but also by a different experience of the body. Experts-by-experience describe themselves as emotionally numb, detached from their bodies and restricted in their movements (Fusar-Poli et al., 2023). The inner and outer world become increasingly distant as the subjective body loses its ability to resonate with its surroundings in the state of depression (Fuchs, 2014). These are not mere subjective accounts; they are reflected in coherent psychophysiological findings. In affective contexts, positive behavioral together with negative physiological reactions tend to be less prevalent in depressed individuals, which complements self-reports pointing in the same direction (Bylsma et al., 2008). The processing of positive stimuli was shown to be altered in affective startle response paradigms (Boecker and Pauli, 2019; Vaidyanathan et al., 2014). Studies also repeatedly found slower gait patterns in depressed individuals (Elkjær et al., 2022). On an interactional level, less interpersonal involvement behavior at the end of treatment may predict the risk of a future recurrence of a depressive episode (Bos et al., 2002; Geerts et al., 2006). In this light, findings regarding differences in the bodily dimension of depressed populations complement subjective accounts. Functional alterations in the connectivity of sensory and motor cortices coinciding with depression (Ray et al., 2021; Wüthrich et al., 2024) add neural correlates to this phenomenological presentation, which might explain how the subjective experience of depression is modulated by psychomotor processes (Northoff et al., 2021). Having in mind the high prevalence of depressive disorders with an estimated 332 million individuals affected and rising disability adjusted life years (Ferrari et al., 2024), understanding how the interplay between the bodily dimension and the subjective experience of depression can be addressed in psychotherapies is a promising endeavor.

### Subjective bodies in psychodynamic psychotherapies

Directing the attention towards psychodynamic psychotherapies for depression, we surprisingly find very limited systematic recognition of the bodily dimension. A glimpse at effective depression treatments alone could motivate attending to the bodily dimension, where behavioral activation was repeatedly found to have large effects on depressive symptomatology (Cuijpers et al., 2020, 2023). One possibility for psychodynamic psychotherapies could simply be the integration of these effective interventions (Shahar, 2021). Yet, integrative efforts come with their own challenges since a theoretical fit must be reestablished for every psychodynamic theory tradition, we want the borrowed intervention to be applied in. Certainly there are also authors within psychodynamic grounds advocating for the use of an embodied understanding and technique (Broschmann and Fuchs, 2020; Doering, 2022; Leikert, 2021; Ogden, 1994; Ruettner et al., 2015; Leuzinger-Bohleber, 2017). These ideas nevertheless come with their own limitations, for they mostly find their epistemic foundations in aggregated single cases. Despite this, shared theoretical principles allow for newly developed interventions to be applied with relative ease within other psychodynamic theory traditions. The most fundamental commonality we find is an inward focused attention on bodily processes happening within the patient. Sometimes this position is further extended to signals from the analysts’ body and therein a countertransference within the analyst’s subjective body. In accordance with this view, the Boston Change Process Study Group (2018) suggests using the term *moving through* instead of *working through* to highlight the process happening between the individuals involved in the therapeutic endeavor and their subjectively experienced bodies. In particular, they draw attention to the process of mutual understanding, governed by reciprocal identificatory processes, which are also informed by nonverbal communications, albeit not exclusively.

While the former approaches can be thought of as inductive in terms of their theory building when it comes to embodied practices, other authors harnessed findings from neuroscience and psychotherapy research to integrate the subjective body. In an attempt to augment psychodynamic thinking with a focus on emotions Lane (2020) for example stresses the importance of subjective bodily experiences for reorganizing emotional relating towards others. The model encourages the exploration of states observed within oneself and within others in a hierarchy of ever more complex emotional experiences to achieve this goal (Lane and Smith, 2021). It is important to note that bodily sensations in this theory form the foundation for more nuanced experiences, that at the highest level of the hierarchy allow for differentiated feelings to be attributed to the self and others.

Observable bodily phenomena as well as bodily experiences are also readily used in Intensive Short Term Dynamic Therapy (ISTDP), which was developed based on extensive video-based microprocess research (Abbass et al., 2013). In this particular treatment model bodily cues serve as a diagnostic marker and as a cornerstone of interventions aiming at connecting behavioral with emotional phenomena (Abbass, 2016). Bringing together the models mentioned thus far, they integrate observations of the patient’s and of the therapist’s body with subjective states experienced by the patient or within the dyad. These theoretical frameworks are therefore in line with a dual aspect monism (Solms, 2021a) rooted in findings on the hierarchical organization of the brain (Panksepp et al., 2017; Solms and Friston, 2018). They acknowledge a physical reality in a shared intersubjective space and a subjective experience, which may not be in congruence. Essentially, what they do, even though not explicitly, is to introduce the idea of a lived body or in condensed German: *Leib.* Böhme (2020) conceptualizes the *lived body* as an objective body, as it is observed in medical research for example, that by demands made upon this body *can* be experienced. Drawing on this definition, but highlighting the intersubjective nature of psychotherapy, we will use the term *subjective body* to denote a body that is made available to subjective experience by therapeutic interventions. But does anything change once we address the subjective body in the process of psychotherapy, and does it add value to the psychotherapeutic process from an empirical viewpoint?

### Existing evidence

Experiential dynamic therapies are a specific type of psychodynamic treatment that strives to facilitate affective processing while making it an indicator of the therapeutic process. In a meta-analysis (Lilliengren et al., 2016), experiential dynamic therapies were compared to other bona fide therapies regarding general psychiatric symptoms. The findings were compiled across various psychopathologies. No differences were found post-treatment. However, the follow-up outcome favored experiential dynamic therapies [*d* = 0.75, 95% CI (0.18, 1.32), *p* = 0.010], as they subsequently showed better results than the bona fide comparators. A subgroup analysis found the same relationship for depression. While some experiential dynamic therapies address bodily cues alongside subjective experiences, this is not always the case. ISTDP (Davanloo, 2000), for example, is a type of experiential dynamic therapy that incorporates techniques for addressing the subjective body, as outlined previously. Malan’s approach is also subsumed under this theoretical framework, but does not always relate affectivity to bodily processes (see Malan, 1963 as opposed to Malan, 1986). This makes it difficult to directly infer a conclusion on the efficacy of psychodynamic psychotherapies addressing the subjective body in general and in depressed populations in particular.

Furthermore, findings regarding affect or emotion focus do not converge. In an earlier meta-analysis, psychotherapies focusing on affect were associated with a medium-large effect (Diener et al., 2007) compared to therapies without such a focus. It is unclear whether the bodily dimension was related to the subjective experience in the included studies, as the focus of the interventions was on the experience *or* expression of current feelings. In another meta-analysis, using a focus on emotions or interpretative interventions among psychodynamic therapies as a binary moderator, a weaker effect was found for treatments focused on emotions (Driessen et al., 2015). However, these were exploratory findings, and only one study was identified as emotion-focused compared with 14 categorized as interpretative.

At this point, it is not clear how interventions addressing the subjective body in verbal psychodynamic psychotherapy influence the treatment effect directly. Existing studies will only allow an insight into the average efficacy of psychodynamic treatments that do address the subjective body in depression. One way to illuminate this topic is to identify studies that included relevant interventions. In this context, manuals or intervention descriptions are the foundation on which further conceptualizations can be built. The individuality of case formulation in psychodynamic therapies makes such a manualization challenging, but not impossible (e.g., Luborsky, 1984; Strupp and Binder, 1984; Taylor, 2015). Manuals themselves may introduce new difficulties in causal inference through what could easily be called a “package problem” (Tolin et al., 2015). Specific interventions are part of a greater set of interventions comprising a package which is embedded in a theoretical framework. We simply do not know how single interventions influence the outcomes, unless we leave out or add specific interventions and compare them to the treatment without the modification. Another challenge when including interventions addressing the subjective bodily experience in the interventional repertoire, is that these treatment components must be formulated clearly. Examining treatment manuals used in randomized controlled trials (RCT) on depression, such as those mentioned previously (Luborsky, 1984; Strupp and Binder, 1984; Taylor, 2015), revealed a lack of such explications. This study therefore poses two questions: how are subjective bodies verbally addressed in psychodynamic depression treatments and what is the average efficacy of those treatments? The effects of psychodynamic treatments addressing the subjective body are hypothesized to: (H1) differ from those of control conditions and (H2) show equivalence to bona fide therapies (BFT).

## Methods

### Pre-registration

This systematic review was prospectively registered with PROSPERO (CRD42024523337).

### Literature search

A combined search strategy was applied to identify relevant literature. We searched the databases PsychInfo and Medline using terms related to psychodynamic theory, intervention, embodiment, depression, and randomized study design connected by Boolean operators (see supplemental material for details). Results were limited to journal articles written in English or German. Rayyan (Ouzzani et al., 2016) was used to organize the review process. Duplicates were removed with its inbuilt autoresolver, setting the similarity of articles to a minimum of 97% also requiring matching DOIs. Inclusion followed a two-stage procedure and was conducted by two trained and supervised undergraduate students (A. A and V. S.). Conflicting ratings were resolved by discussion with the first author (M. H.). An initial search was conducted in March 2024 and was rerun in January 2025 for publications till December 2024. Reporting of findings follows the PRISMA 2020 Statement (Page et al., 2021).

Articles were first inspected based on their titles and abstracts. Included articles all had to report outcomes of RCTs on depressive symptomatology where at least one intervention was based on psychodynamic theory. Additional literature was identified in a frequently updated list of psychodynamic RCTs (Lilliengren, 2023) as well as in recent meta-analyses (Barber et al., 2021; Smith and Hewitt, 2024; Woll and Schönbrodt, 2020) targeting the efficacy of psychodynamic interventions. In the second stage, the full set of inclusion and exclusion criteria was applied to the full texts, which were organized using the PICO scheme (Higgins et al., 2024). Studies were eligible if they reported on populations of depressed adults (over 18 years old) and used a recognized measure of depression (e.g., HDRS, BDI, MADRS) as an inclusion criterion. Studies on children and adolescents as well as those not targeting depressive symptoms were excluded. At least one intervention in the trial had to be individual verbal psychotherapy addressing the subjective body during the psychotherapy process and needed to be based on psychodynamic theory, as defined in the study itself or a referenced manual. Where the intervention description was not publicly available authors were contacted at least twice and asked to provide a copy of the manual used in the study. To fit the definition of addressing the subjective body the intervention description or manuals had to contain techniques or explain in examples, how a connection between observed bodily phenomena and subjective experience of the patient is made in the treatment model. Treatment descriptions were double coded to ensure the interventions met this description and conflicting ratings were discussed in the team. Table 1 illustrates examples of manuals we in- and excluded in this process.

**Table 1:** In- and exclusion of studies addressing the subjective body.

Type of psychotherapy	Example from treatment description	Explanation of decision
Excluded		
Supportive expressive therapy (Luborsky, 1984)	Principle 1 “Understanding the Symptoms in the Context of Relationships” (p. 94) introduces the concept to “…focus understanding around symptoms and related affects.”(p. 95). The meaning of affect is briefly mentioned as referring to “most often anxiety and depression” (p. 95). A therapist’s intervention based on this principle is illustrated: “Each time I notice and comment that you are looking attractive or that you are doing well in your work you get tearful and cry.” (p. 96)	Affect is operationalized broadly, followed by a vague idea about how affects that are showing in the body can be addressed. Based on the example it also becomes clearer that the bodily dimension is referred to but it is not connected to the subjective experience of the patient. This approach aligns therefore more closely with a generic focus on affect.
Included		
Intensive short term psychodynamic psychotherapy (Abbass, 2015)	Among other interventions addressing the body, the technique of “Pressure” (p. 70) is used to understand what is felt and how the feeling is experienced physically. The excerpt of a session makes this idea accessible: “Pt: (Appears very tense; clenches hands and sighs.) Th: I noticed when you came in that you are tense. Do you notice that? (Pressure to be conscious of anxiety.) Pt: (Smiles slightly and sits up straight.) Yeah, I get tense and I get sore muscles. Th: Can we look at what feelings create that tension? What feelings do you have here with me? (Pressure to identify feelings.) Pt: (Smiles and sighs.) Well, I was a little bit nervous about coming in. (Tactical defenses and striated muscle tension.)” (p.57)	The study using this manual was included, because observed information about the body is first verbalized, then clarified, and thereby made available to reflection. Importantly, the information gained is then explicitly elaborated on with respect to the subjective experience of the patient.

Comparators were either bona fide psychotherapies (e.g., CBT, Interpersonal Psychotherapy or Systemic) or control groups (treatment as usual, placebo, waiting list). Studies comparing psychodynamic therapy without pharmacotherapy to the administration of pharmacotherapy were excluded. Outcomes relevant for analysis were depressive psychopathology at end of treatment and follow-up measured by an established criterion (e.g., HDRS, BDI, MADRS). Additionally, interpersonal functioning (e.g., IIP) was considered as a secondary outcome, as it provides information about an important aspect of overall functioning and allows for a more detailed analysis of the treatments included.

### Data extraction and analysis

After the searches were finished, the first author (M. H.) and one trained student assistant (A. A.) extracted the data. Information relevant to the study was the mean and standard deviation of interventions post-treatment and on longest follow-up period, together with their respective sample sizes. Where this information was lacking, standardized mean differences (SMD) were calculated from the most comprehensive available data. If data was available on more than one relevant outcome, effects were aggregated within trials (Higgins et al., 2024), assuming high correlations = 0.80 (Bukumiric et al., 2016) between outcomes at posttreatment and thereafter. Treatments with different durations but the same intervention manuals were also aggregated (Higgins et al., 2024). Further variables relevant were year of publication, treatment conditions, sex, type of analysis, number of sessions, duration of treatment, and time till follow-up. Described or referenced psychodynamic treatment models were extracted from included articles by the first author (M. H.) as part of the review procedure. The identified treatment models were then summarized narratively and clustered by similarity of their theoretical background.

Quantitative synthesis was performed by applying random-effects models to the data using the “metafor” package (Viechtbauer, 2010) in R version 4.5. (R Core Team, 2025), since at least moderate heterogeneity was expected (Driessen et al., 2015; Leichsenring et al., 2023). Effect sizes (Hedges’ g) were calculated and aggregated before applying separate analysis for BFT and control group as comparators to psychodynamic interventions. The outcomes at end of treatment and at the follow-up were evaluated separately. A restricted maximum likelihood estimator (Viechtbauer, 2005) was used to model heterogeneity in effect estimates. Heterogeneity between studies was reported using the Q statistic and I^2^ (Higgins et al., 2003), where values of 25, 50 and 75% are referred to as low, medium, and high heterogeneity, respectively. All meta-analytic calculations were performed in a manner that allow for the interpretation of negative standardized mean differences as a symptom decrease in favor of psychodynamic treatments, while positive estimates favor comparison conditions. Results are visualized using forest plots. To test against BFT, two-one-sided-tests were applied, as implemented in the “TOSTER” package (Caldwell, 2022). This acknowledged the deficit of null hypothesis significance testing when comparing psychotherapeutic interventions regarding their equivalence (Lakens, 2017), for the absence of a difference in a two-sided test logic cannot be equated with equivalence. The TOSTER package used data from a previously specified random-effects model as input. The upper and lower bounds for the two-one-sided-tests were set to a minimal important difference of 0.24 standardized mean differences from zero (Cuijpers et al., 2014), which represents a relief in symptom burden just large enough to be subjectively experienced by patients.

Sensitivity analysis consisted of influence diagnostics via a leave-one-out analysis (Viechtbauer, 2010; Viechtbauer and Cheung, 2010) to detect studies affecting overall effect estimates. We inspected potential publication bias by applying a regression test to the fitted models. Where potential threats to the validity of the findings were detected, the parameters of the models without influential studies are reported. Potential moderators were tested to see whether effect sizes varied as a function of study characteristics.

### Quality assessment of studies

All included studies were rated using the RCT of Psychotherapy Quality Rating Scale (RCT-PQRS) developed by Kocsis et al. (2010). The scale comprises 25 items, with the first 24 asking for specific properties of the study on a scale from 0 to 2, and item 25 asking about the overall quality on a scale ranging from 1 to 7. A study must reach a score of 24 or greater, to be considered “reasonably well done” (Gerber et al., 2011, p. 24). In this case all of the quality criteria must be implemented sufficiently, or some criteria are met at a high standard while others do not need to be met at all. In addition to asking about the study’s generally relevant design features, it also focuses on the psychotherapy actually delivered in the context of the rated study, as well as the reporting of applied procedures. This is important because a major deviation from a given manual makes causal inference regarding the intended treatment spurious (Leichsenring et al., 2017), as previously pointed out. Ratings were carried out by the first author (M. H.) and a trained student (A. A.). Both underwent training on a set of non-included RCTs until reasonable agreement was reached. Calculating the intraclass correlation on the sample included in the meta-analysis showed an ICC (3.1) = 0.83 (Koo and Mae, 2016; Shrout and Fleiss, 1979) meaning agreement was good.

## Results

In an initial search 10.333 articles were identified (PsychInfo: 5.495 articles & Medline: 4.838 articles). After removing 2.116 duplicates, 8.217 articles were screened. This search resulted in 25 articles remaining for full-text search. Out of Lilliengren (2023) list of psychodynamic RCTs 72 additional studies were identified from all studies focusing on depression or mixed samples. Data of one follow-up study was included as well. Searching in reference lists of meta-analyses and articles included did not yield any new studies after all. No manual was available from one single study (Jakobsen et al., 2014) which led to exclusion for a lack of information.

**Figure 1:**
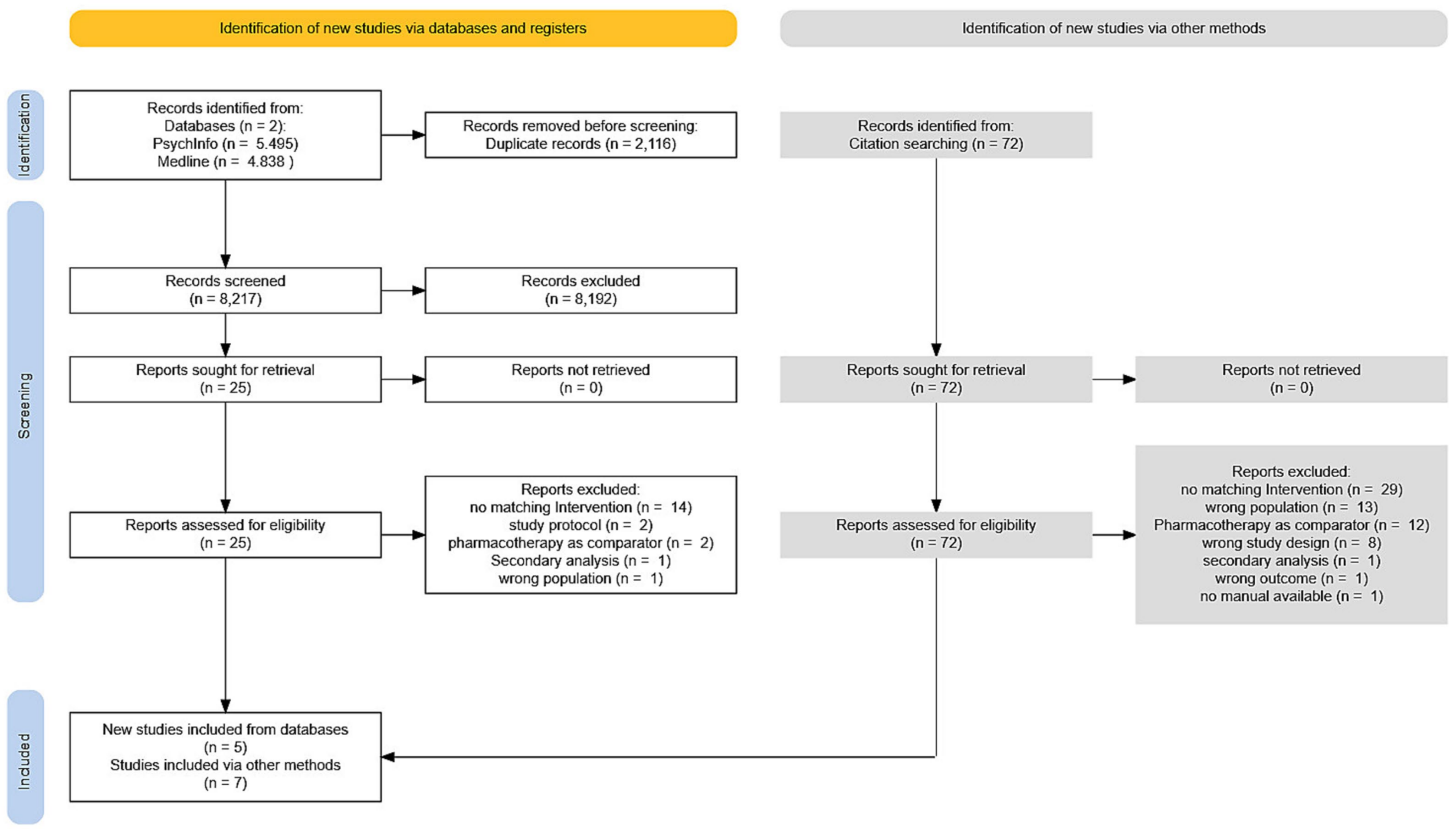
PRISMA flow diagram.

Full-text search resulted in *n* = 11 trials (Ajilchi et al., 2016; Barkham et al., 1999; Driessen et al., 2013; Fonagy et al., 2020; Gibbons et al., 2012; Herrmann-Lingen et al., 2016; Heshmati et al., 2023; Meganck et al., 2023; Shapiro et al., 1994; Town et al., 2017a; Wang et al., 2023) and one follow-up report (Town et al., 2020) remaining in the final sample (for details, see Figure 1).

Study characteristics are summarized in Table 2. Quality ratings of all included studies were above 24 points (range = 32–44.5) indicating that they were at least reasonably well executed (for details, see Supplementary Table S1).

**Table 2:** Characteristics of studies included.

Author	Year	PDP approach^1^	Comparator^1^	Main ouctcome^2^	n _PDP_	n _Comparator_	Number of sessions intended	Treatment duration in weeks	Follow-up	%female	Manual^1^
Control											
Ajilchi et al.	2016	ISTDP	WL	BDI-II	16	16	15	NA	yes	28.13	ISTDP (Abbass, 2015)
Fonagy et al.	2020	DIT	LIT, CBT	HRSD/BDI-II	73	54	18	24	no	65.99	DIT (Lemma et al., 2010)
Gibbons et al.	2012	BDP	TAU	HDRS	16	13	12	12	no	87.10	AFT (Crits-Christoph et al., 2006)
Herrmann-Lingen et al.	2016	SI-SET	TAU	HADS-D	284	285	3	4–6	no	21.05	SPIRR-CAD-Manual (Fritzsche et al., 2011)
Heshmati et al.	2023	ISTDP	WL	WAI-Depression	36	39	20	NA	yes	61.63	ISTDP (Davanloo, 2000; Schröder et al., 2015)
Town et al.	2017/2020	ISTDP	TAU	HRSD/PHQ-9	30	30	20	NA	yes	65.00	ISTDP (Abbass, 2015; Davanloo, 2000)
Wang Y et al.	2023	DIT	GST	HRSD/PHQ-9	66	75	16	16	yes	77.30	DIT (Lemma et al., 2011b)
Bona fide therapy											
Barkham et al.	1999	PIT	CBT	BDI	54	62	3	14	no	42.24	Conversational Model (Hobson, 1985)
Driessen et al.	2013	SPSP	CBT	HDRS/IDS-SR	177	164	16	22	yes	70.09	SPSP (de Jonghe, 2005)
Meganck et al.	2023	STPP	CBT	HRSD	38	39	20	NA	no	68.00	UPP (Leichsenring and Schauenburg, 2014)
Shapiro et al.	1994	PIT	CBT	BDI	28 + 29	29 + 29	8/16	NA	no	52.14	Conversational Model (Hobson, 1985)

^1^BDT, Brief Dynamic Psychotherapy; CBT, Cognitive Behavioral Psychotherapy; DIT, Dynamic Interpersonal Therapy; GST, General Supportive Therapy; ISTDP, Intensive Short Term Dynamic Psychotherapy; LIT, Low Intensity Treatment; PIT, Psychodynamic Interpersonal Therapy; SI-SET, Stepwise Individual Supportive Expressie Therapy; SPSP, Short-term Psychoanalytic Supportive Psychotherapy; STPP, Short Term Psychodynamic Psychotherapy; WL, Wait List; TAU, Treatment as Usual; ^2^BDI, Beck Depression Inventory (II); HADS-D, Hospital Anxiety and Depression Scale- Depression; HRSD, Hamilton Rating Scale for Depression; IDS-SR, Inventory of Depressive Symptomatology Self-Report; PHQ-9, Patient Health Questionnaire 9; WAI-D, Weinberg Adjustment Ineventory-Depression Subscal.

### Psychodynamic psychotherapy vs. control conditions

**Figure 2:**
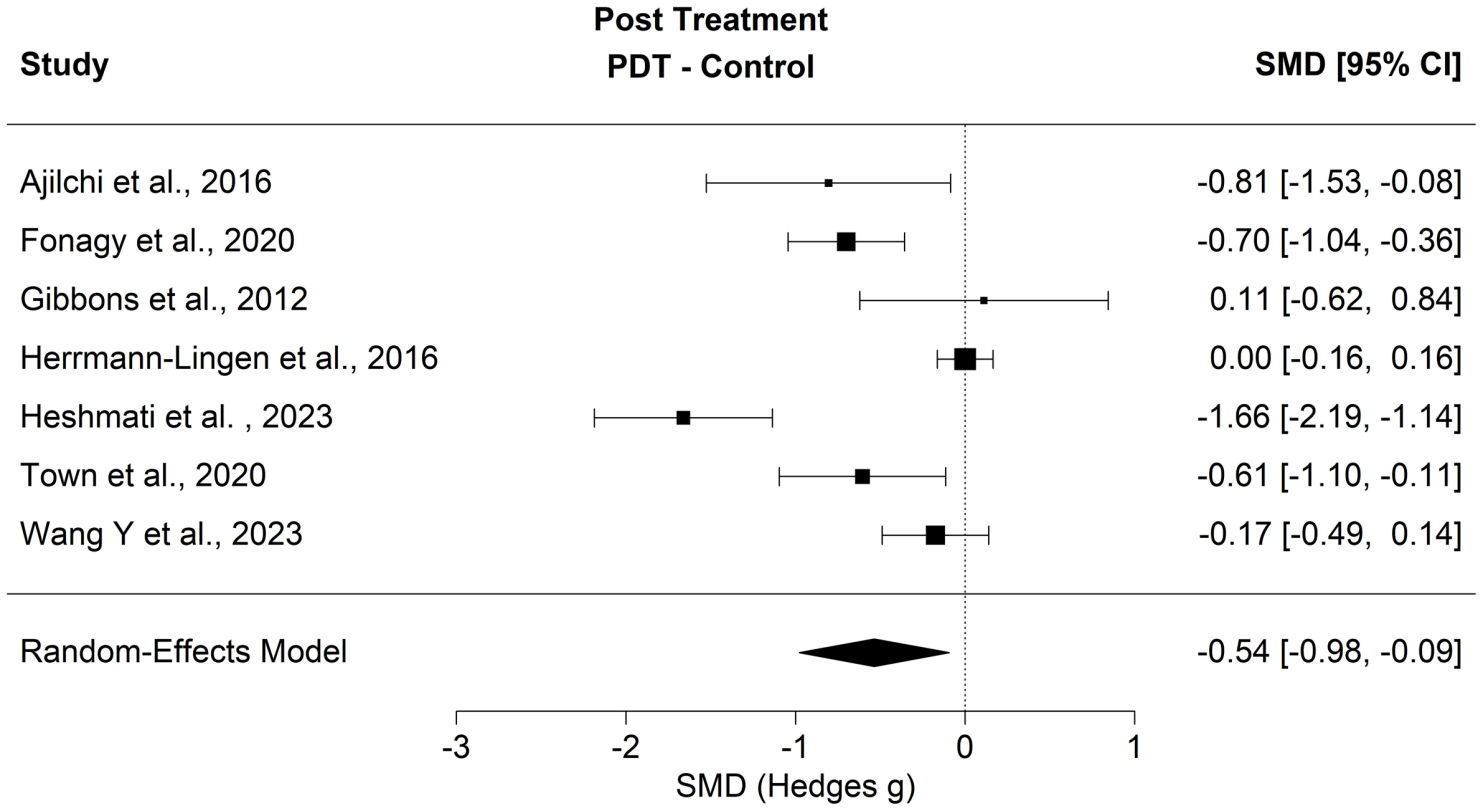
Forest plot comparing PDP to control groups post-treatment.

Comparisons of psychodynamic treatments addressing the subjective body to control conditions were found in seven trials including a total of 1,032 individuals studied. The full model estimated the effect at standardized mean difference of SMD = −0.536 [*p* = 0.017, 95% CI (−0.98, −0.09), *k* = 7] in the presence of high heterogeneity (*Q* = 48.62, *I*^2^ = 89.12%; see Figure 2). Funnel asymmetry was not detected in this context as indexed by a regression test (*z* = −0.66, *p* = 0.506). Sensitivity analysis revealed two influential studies. One study (Heshmati et al., 2023) showed a large effect estimate for symptom reduction. Its exclusion reduced heterogeneity to a moderate level (*Q* = 19.80, *I*^2^ = 72.49%) changing the difference between interventions to a standardized mean difference of SMD = −0.33 [*p* = 0.026, 95% CI (−0.63, −0.03), *k* = 6]. After exclusion of another study (Herrmann-Lingen et al., 2016) heterogeneity settled at a moderate level (*Q* = 8.53, *I*^2^ = 50.72%) again changing the models estimate to SMD = −0.44 [*p* = 0.003, 95% CI (−0.74, −0.14), *k* = 5]. Removing these studies reduced heterogeneity at the same time the strength of the effect found varied with the inclusion of single studies. Yet there was no overlap of the estimated confidence intervals with zero. We also tested the model with *k* = 7 studies for moderating variables (see Supplementary Table S2). Year of publication (*p* = 0.13) or PQRS-ratings (*p* = 0.31) did not moderate the effect whereas the number of treatment sessions intended in the respective studies did have a moderating effect (*b* = −0.062, *p* = 0.043).

**Figure 3:**
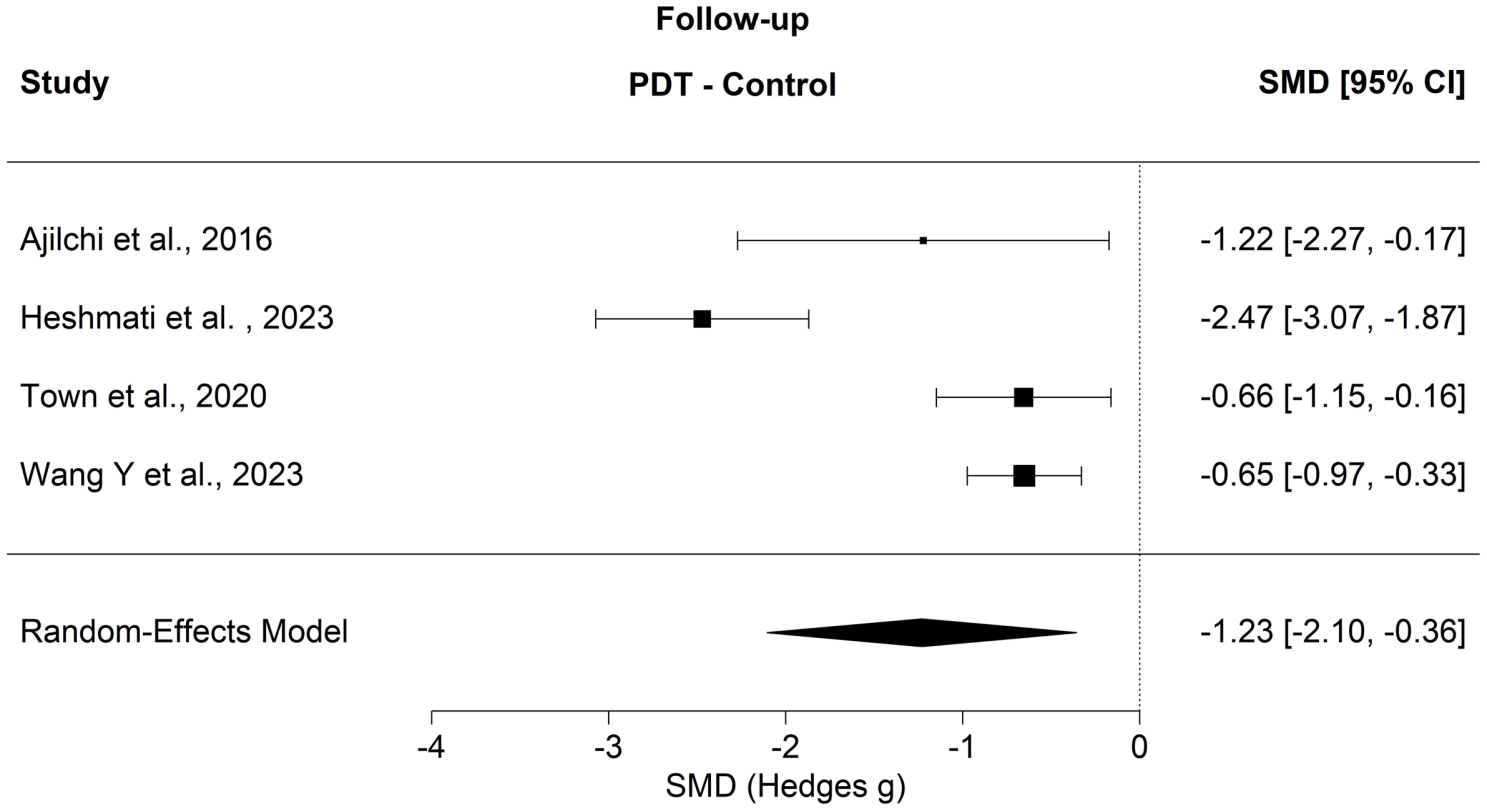
Forest plot comparing PDP to control groups at follow-up.

The long-term effects of psychodynamic treatments addressing the subjective body were evaluated in four trials. On average, 42 weeks (sd = 20) passed from the end of treatment to the evaluation of the follow-up outcomes. Aggregated results show that standardized means were estimated to differ by SMD = −1.23 [*p* = 0.005, 95% CI (−2.10, −0.36); see Figure 3]. Funnel plot asymmetry was not detected (*z* = −0.4583, *p* = 0.6468). Heterogeneity of findings (*Q* = 29.4758, *I*^2^ = 90.35%) nevertheless was high.

### Psychodynamic psychotherapy vs. bona fide therapy

**Figure 4:**
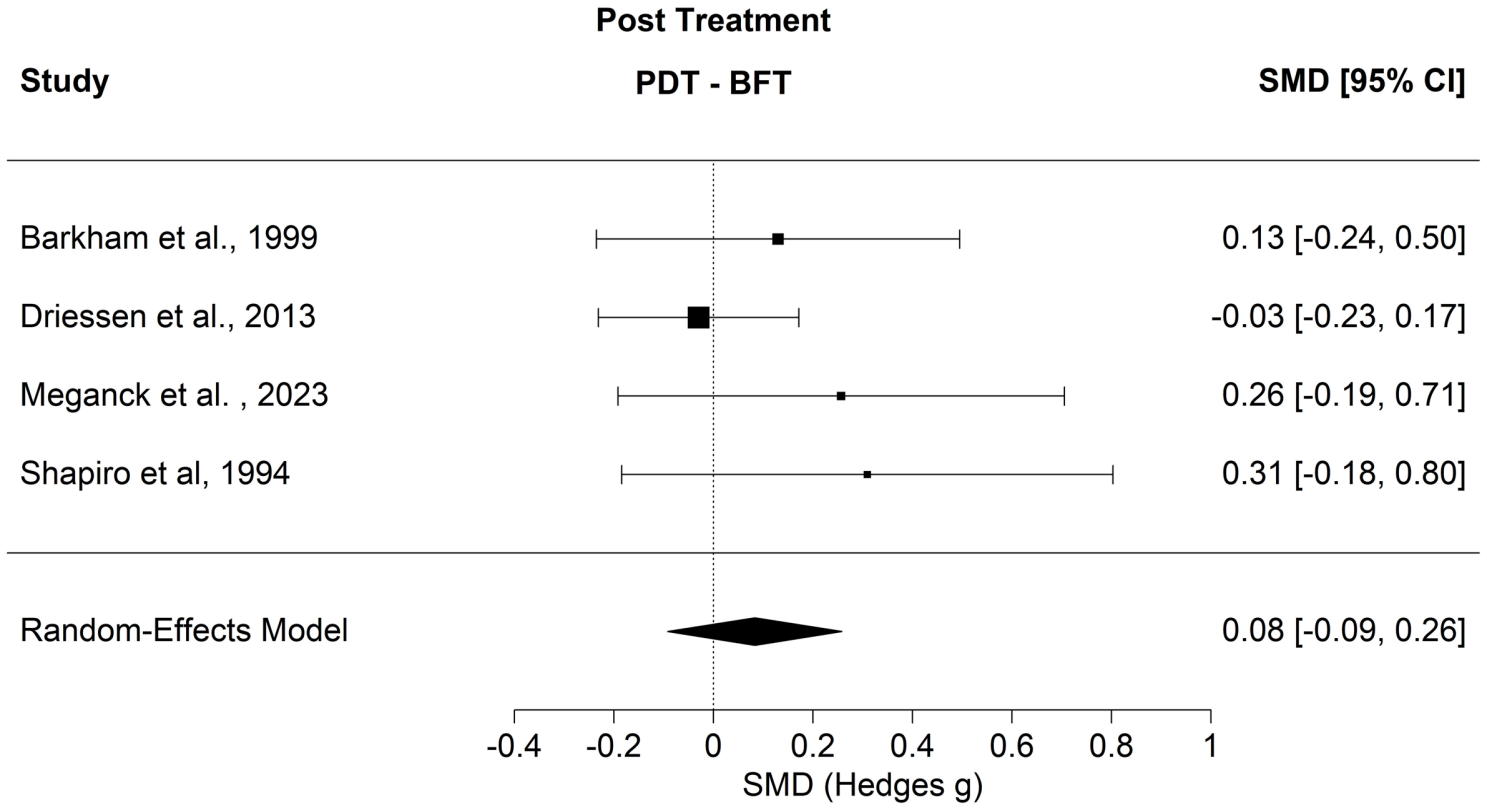
Forest plot comparing PDP to BFT.

A total of four studies including a total of 592 individuals met the inclusion criteria for a comparison of the efficacy of psychodynamic treatments addressing the subjective body with that of other bona fide therapies. Comparators were cognitive behavioral therapies in all four trials (see Table 1). Results at the end of the treatment showed a SMD = 0.08 [*p* = 0.352, 95% CI (−0.09, 0.26)]. In this context, heterogeneity seemed to have had little influence on the results (*Q* = 2.6125, *I*^2^ = 11.68%). Estimated model parameters were then evaluated with two one-sided tests. The confidence interval of the estimate did not exceed a clinically relevant effect of SMD = 0.24 [*z* = −1.756, *p* = 0.039, 90% CI (−0.064, 0.23)]. Sensitivity analysis indicated no influential studies. At the same time, no funnel plot asymmetry was present (*z* = 1.61, *p* = 0.107, for details, see Figure 4).

Follow-up data was unavailable for k≥3 studies; therefore, no results can be reported. One study (Fonagy et al., 2020) also compared a psychodynamic treatment to a bona fide treatment. However, it was not included in the meta-analysis since the authors stated that they did not have sufficient statistical power to test for equivalence to CBT due to unequal sample sizes of the treatment arms. Rather, it was intended as a benchmark of acceptability given the novelty of the treatment studied.

### Approaches minding the subjective body

Throughout the included studies 10 manuals were referred to. They present with a multitude of theoretical foundations derived from a dynamic understanding of the human psyche. We will briefly present central ideas regarding the use of interventions aimed at a subjective understanding of nonverbal bodily processes shared in the intersubjective space. Since some trials originate from similar theoretical models, they will be presented together.

Overlap was found regarding the use of ISTDP, as it was subject of three trials (Ajilchi et al., 2016; Heshmati et al., 2023; Town et al., 2017b; Town et al., 2020). These works all drew on manuals describing a single treatment approach with a shared theoretical foundation, even though in different shades of it is development (Abbass, 2015; Davanloo, 2000; Schröder et al., 2015). Their main goal is an “unlocking of the unconscious” (Davanloo, 2000, p. 8) via the experience of previously suppressed feelings. ISTDP consists of specific intervention phases, namely: inquiry, pressure, challenge, head-on collision and access to the unconscious, afterwards unconscious material is integrated (Davanloo, 2000). Phases are observed to vary between individuals. Four major ideas about nonverbal information are formulated in this work. Firstly, bodily cues like avoiding eye contact, deep sighs or stiffness in posture are vehicles in the phases of pressure and challenge to point out defenses and make propositions regarding the affective state currently activated. Another more subtle path is taken when therapists assess exactly how a present feeling is experienced to help the patient verbalize information going beyond the cognitive representation of a feeling (Davanloo, 2000, p. 199). A third idea evolves around two distinct somatic pathways (Davanloo, 2000) for anxiety and rage. For example, striated muscle activity starting in the hands and forearms, spreading to the core region of the body, finally ending in the legs, is a distinct cascade of physiological reactions used as a diagnostic marker of anxiety. These two somatic pathways help to formulate hypotheses about the patient’s feelings grounded in a visually observable correlate. A fourth mechanism was identified with regard to the appearance of breaking through to the unconscious material. Here a loss of tension in striated muscle tissue accompanied by the appearance of sadness and a reduction in unconscious anxiety are theorized to *always* coincide with the emergence of unconscious material.

Abbass (2015) further differentiates between activation of smooth and striated muscle tissue to guide the therapy process. Smooth muscle activity is associated with repression of unconscious anxiety and lower structural capacities. Defenses leading to activity in smooth muscles can manifest, for example, as an irritable bowel syndrome or coughing. This is a clear sign to change towards observing the affective and bodily processes together with the patient. Repeated work on this processing mechanism leads towards activity of striated muscles with ever greater capacity to tolerate confrontation. The author gives a dense description of this process when describing it as “emotionally activated study of body responses” (Abbass, 2015, p. 195). Intermittent overstimulation is theorized to again lead to a loss of tension in striated muscle tissue. Following this theory observable muscle activation gives a clear indication of the extent to which the patient can tolerate confrontation. Whenever the total absence of striated muscle activity is observed this should be explored in detail. When no signaling takes place, a range of possible mechanisms need to be taken into consideration. Denial, primitive defense mechanisms, passivity or hopelessness are among the mechanisms described to prevent signaling (Abbass, 2015). The clinicians’ experience in this regard is referred to as instructive in identifying what prevents a dynamic process from developing. Experience once more is demanded when therapists attend to their own empathic resonance, using their bodily sensations to generate hypotheses about their patient’s current feelings (Abbass, 2015, p. 141). No further extension of these principles is to be found in the work of Schröder et al. (2015). The somatic component of emotional experience throughout all works is emphasized as one of the main factors leading to change.

Two other studies applied Dynamic interpersonal Therapy (DIT) (Fonagy et al., 2020; Wang et al., 2023) again using fairly similar references (Lemma et al., 2010; Lemma et al., 2011a; Lemma et al., 2011b). DIT is a time-limited treatment centered around an interpersonal affective focus (IPAF). Affects are seen as the link between self and others in forming recurring interpersonal schemes. Bodily cues here are vehicles to access unconscious feelings. While the IPAF serves as the main pillar, bodily cues facilitate focus building as the process unfolds. This surely needs further elaboration. In DIT information displayed divergently between the verbal and nonverbal level can be targeted by interpretations, always trying to bridge these observations with the sessions IPAF (Lemma et al., 2011a). Suitable nonverbal expressions can also be addressed to explore the patient’s subjective experience thoroughly. Defenses hindering integration of different levels of communication are challenged. “Hyperembodiment” (Lemma et al., 2010, p. 336) on the other hand is associated with a loss of mentalizing capacities where mental and physical representations become hardly divisible. Symbols are then embodied rather than being mental entities, thus making it again necessary to challenge overly unflexible representations by remaining curious about the patient’s mental states in light of the bodily representations.

Building on Supportive Expressive Therapy and adding components where necessary three studies (Gibbons et al., 2012; Herrmann-Lingen et al., 2016; Meganck et al., 2023) complemented existing manuals to fit their researched populations needs. Psychodynamic interventions in one study (Herrmann-Lingen et al., 2016) aimed at improving depressive symptomatology in patients with coronary artery disease. Individual therapy was part of a stepped care plan in this study and consisted of three sessions. Psycho-somatic links are sought for in the patient’s current somatic complaints as well as narratives of the patient’s past and present experiences, connecting them to their affective representation. Nonverbal displays of emotion are considered as indicators helping to formulate hypothesis about the patient’s current affective state. Moreover, inconsistencies in emotional reactions are pointed out, that is a divergence between nonverbal displays of emotion and content reported in-session. Interventions therefore vary between clarifying and confronting unconscious affective states that are subject to the patient’s defenses. Therapists are however encouraged to leave open their hypothesis to correction by the patients.

Adapting and furthering a supportive expressive framework Gibbons et al. (2012) and Meganck et al. (2023) eventually refer to Alliance-Fostering-Therapy (Crits-Christoph et al., 2006). Here we find a focus on ruptures where nonverbal cues are indicative of a breakdown in the therapeutic relationship. Addressing these ruptures by means of underlying affect allows working through conflicts. Interventions could for example connect nonverbal behavior like avoiding eye contact with the overall process. Another aspect is focusing on the therapist’s own nonverbal communication by trying to show affiliative behavior in posture and facial expressions. Meganck et al. (2023) also refers to the unified psychodynamic protocol for depressive disorders (Leichsenring and Schauenburg, 2014) as one of their main pillars for the studies manual. Here again an emphasis is laid on helping the patients observe bodily sensations accompanying emotions and affects. The concept does not extend beyond this short mention.

Hobsons conversational model (1985) is applied in two trials (Barkham et al., 1999; Shapiro et al., 1994). Nonverbal cues are followed closely to infer general arousal, circumscribed emotions, or hypotheses about manifest feelings. Clear differences, however, exist in the extent of attention paid to the therapists’ own bodily involvement. The attention is alternately directed towards the patient and the therapist herself, going back and forth between those perspectives. Nonverbal cues of both parties are observed on a moment-to-moment basis. Symbols emerging in the dialogue, and the body here is seen as a symbol carrying subjective meaning for both parties involved, need to be processed in a joint endeavor towards their relevance for the here and now. This is where the repetitive relational patterns are enacted according to the theory. From a technical standpoint the conversational model encourages the use of bodily terms in interpretations, that is using feeling words, referring to the patients’ or the therapists’ subjective bodily experiences. Direct mappings from symbols to meanings are not promoted. They have to be individually (re-)constructed as the therapeutic process evolves. Every aspect of therapy is seen under the lens of continuous “mutual correction of misunderstandings” (Hobson, 1985, p. 196). This extends also to cues picked up through nonverbal behavior. By working through the various symbols that occur along the process, contextualizing them with the experiences they are accompanied by, symbolization is striven for, meaning that more abstract dynamics become represented in the patients’ thinking.

Another study (Driessen et al., 2013) compared therapists trained in Short-term Psychoanalytic Supportive Psychotherapy (de Jonghe, 2005) with those trained in cognitive behavioral therapy. It is acknowledged that nonverbal communication is important to this approach. Yet, the extent of explication is limited. Interventions aiming at clarification, for example, are directed at verbal and nonverbal communications in order to engage in a more thorough understanding of the patient’s situation. Also, nonverbal displays lacking congruence with verbal contents are used in confrontation, to shed light on dissonances between different levels of representation. Varying levels of experience are therefore connected through attentive inquiries by the therapist. The focus of attention is shifted to nonverbal phenomena presumably unrecognized otherwise.

## Discussion

Currently there are multiple theoretical models at hand (Broschmann and Fuchs, 2020; Leikert, 2021; Ramseyer, 2022) that integrate subjective bodily experiences with the process of the psychotherapeutic dyad. Evidence on the efficacy of treatments applying such models in the context of depression is on the other hand sparse. This review identified 11 trials with 10 psychodynamic interventions referenced that do take into account the subjective body systematically. From these trials in total 1,624 individuals contributed to the results reported here. Findings derived from comparisons of psychodynamic psychotherapies addressing the subjective body to control conditions were promising. Confidence intervals of the average effects found in the reported comparisons to control conditions did not overlap with zero. Based on the results of the random effects model the hypothesis (H1) of a non-zero effects was not rejected. Yet conclusions can only be tentative given the small number of studies contributing to the findings. This is also reflected by the sensitivity analysis that revealed the impact of single studies on the size of the effect estimate. Effects at follow-up were more pronounced in favor of psychodynamic treatments addressing the subjective body with the lower bound of the confidence interval surpassing a clinically meaningful effect of SMD ≥ 0.24 (Cuijpers et al., 2014) on depressive symptomatology. This is a trend already observed in one of the previous studies (Lilliengren et al., 2016). But again, more studies are needed to ensure those findings aren’t spurious. These preliminary results also raise the question of how these gains occur during the follow-up intervals. Another aim of this review was to investigate the equivalence of treatments. This is particularly important due to its relevance in both research and practice. If approaches minding the subjective body violate the assumption of equivalence this could potentially point toward a risk of adverse effects, i.e., preventing patients from achieving the most favorable outcomes. According to the two-one-sided-tests procedure applied in our analysis, we can assume the equivalence of psychodynamic treatments addressing the subjective body and bona fide therapies when considering depressive symptomatology as the outcome. On basis of this result, the second hypothesis (H2) was not rejected. Robustness of findings from a sample of four studies should nevertheless be questioned.

Taken together, the existing evidence at least preliminarily suggests the efficacy of the researched psychodynamic interventions. Equally as important is how the change is accomplished. This review focused only on interventions minding the subjective body. These interventions can be organized into three technical principles:

I. Diagnostic use of bodily phenomena: All manuals included the idea that nonverbal expression is a diagnostic component that provides valuable information about subtle shifts in arousal and mood. This goes hand in hand with exploration and clarification of how the body is experienced. Additionally, in ISTDP (Abbass, 2015) observable muscle activity is used to predict the appropriate intensity of successive interventions.

II. Addressing diverging verbal and nonverbal expressions: Another context in which bodily signals are used involves actively addressing discrepancies between verbal and nonverbal expressions while inquiring about the accompanying subjective state. This occurs in situations where one thing is said but something else is conveyed nonverbally. Clarifying and confrontational interventions are used to propel the process with this information. It is noteworthy that both occurrences happen, something shows in the body rather than in the mind or contrarily a mental representation lacks a bodily representation. This immediately leads us to the third context in which we find references to the body.

III. Connecting bodily and subjective experiences to dynamic processes: Once a preliminary link is made between a bodily and a mental representation, this link is often used to infer psychodynamic processes from it that are then fed back into the process. The ways in which these ideas are fed back to the process differ greatly. Common to these ideas is that nonverbal communications are understood as a form of language, which must be individually and iteratively mapped. Connecting the different bodily and mental representations towards an integrated subjective experience is accomplished by repetitive working through in direction of strengthening structural integration. Single interventions seem to be of little value, much as in psychodynamic therapy overall. Interestingly, we can find some of the ideas put to action by Davanloo (2000) in this regard already in “Studies on Hysteria” (Freud, 1895). Freud describes a rise in (bodily) symptoms proximal to memory traces at the core of a neurosis. Once worked through he then observed a drop in symptomatic tension. It is this central idea around an unconscious connection of bodily with mental processes we still find preserved much later.

Even though there are overarching technical principles in addressing the subjective body in psychodynamic treatment of depression, the impact that they have on the treatment is not clear. Should these interventions modulate the overall effect of the treatments that they are embedded in, there still is uncertainty about what the underlying mechanisms are. As a consequence we tried to integrate the technical principles from the review with existing findings about psychotherapy processes. One of the most well documented process constructs that was found to contribute to therapeutic change is alliance (Flückiger et al., 2018). Building and retaining it requires the capacity to successfully repair ruptures in the therapeutic relationship. A higher frequency of rupture repairs itself on the other hand seems to lead to better outcomes (Safran et al., 2011; Eubanks et al., 2018). It also makes the timely identification of ruptures a major therapeutic task. This is where the first technical principle identified by our review becomes vital since the bodily dimension is diagnostic in the detection of ruptures (Crits-Christoph et al., 2006). Underlining this idea, single case studies on psychodynamic therapy processes already did show associations between attention to bodily processes, ruptures and restoring of the therapeutic alliance (Mylona et al., 2022; Town et al. 2017). It only takes a slight change of perspective to connect the resolution of ruptures to affect regulation as they seem to conceptually overlap. In dyadic affect regulation the exchange of nonverbal information contextualizes the individual’s interpretation of in- and external information. When these implicit nonverbal processes are verbalized, worked on, then normalize and subsequently become implicit again once they are internalized, this might explain the impact of an embodied understanding (Lane and Smith, 2021; Lane et al., 2022; Koole and Tschacher, 2016). It also connects to the second and third technical principle described before, where divergences between bodily expressions and their subjective experience are integrated by directing attention to disparate representations. The connection of the repetitive presence or absence of a specific bodily reaction with biographical material or the transference in the here and now further integrates the information with top-down psychodynamic concepts. Or framed differently, embodied memory traces become available to modification in a new relationship where the emotion regulation is more adaptive (Leuzinger-Bohleber, 2015; Lane et al., 2022; Solms, 2021b). Other technical principles were less coherent throughout the different treatment approaches. They might be thought of as a continuum of individual therapeutic attitudes underlying the various theories. The following section gives a short overview of the conceptual inconsistencies, while maintaining a focus on beneficial practices.

While some authors emphasize the importance of corrections made by the patient (Herrmann-Lingen et al., 2016; Hobson, 1985), others view conclusions as mostly determinate (Abbass, 2015; Davanloo, 2000), even stressing hypotheses repeatedly after they had been rejected by the patient. The degree of epistemic privilege attributed to the therapist thus can be questioned. Arguing in line with the mentalization literature (Fonagy and Allison, 2014) the confidence put into conjectures about observed bodily phenomena should vary as a function of epistemic trust. This also means that therapists need to remain curious about their patient’s subjective experience before formulating strong dynamic hypotheses based on their observations. Moreover, only three manuals discuss the therapist’s own subjective experience of their body (Abbass, 2015; Crits-Christoph et al., 2006; Hobson, 1985), making it a potentially neglected facet of the treatment process. Interventions extend from showing affiliative nonverbal behavior (Crits-Christoph et al., 2006) to the use of empathic resonance phenomena in inferring interpretations (Abbass, 2015; Hobson, 1985). The meager presence of the therapists body throughout the different approaches moreover stands in a stark contrast to the role emotional counter transference reactions generally have in working with the transference and given the long standing theoretical tradition (Heimann, 1950) around the concept. Once we look at the association between negative counter transferences and poorer outcomes, we also realize that the omission of this information might be disadvantageous, especially given the relation found between conter transference management and better outcomes (Dahl et al., 2017; Hayes et al., 2018). A distinction that is also needed for the sake of clarity is between psychodynamic treatments addressing the subjective body and somatic psychotherapy. When comparing the interventions encountered in the review to those of trauma focused somatic psychotherapies (e.g., Curry and Ogden, 2024; Payne et al., 2015), where bodily phenomena are frequently assessed, it becomes evident that psychodynamic approaches do not center around a bottom-up-processing of current experiences, but rather built on top-down processing guided by psychodynamic case formulations when including the bodily dimension.

Another question open to discussion is how the bodily dimension and its subjective experience evolve after a treatment has ended. Our review found that at follow-up differences between psychodynamic treatments addressing the subjective body and control conditions were on average greater than at the end of treatment, pointing towards improvements in symptomatology after the treatment ends. One possible mechanism could be that addressing the subjective bodily dimension helps patients initiate interaction behaviors that regulate mood and interpersonal relationships. This would align well with the lack of initiative in establishing non-verbal synchrony (Paulick et al., 2018), and particularly leading an interaction, which was related to higher depression scores (Jennissen et al., 2024). The impact of interventions addressing the subjective body on the synchrony of nonverbal behavior is yet to be determined.

Returning to the subjective experiences of depressed individuals outlined in the introduction, the application of the three technical principles identified could potentially allow them to reengage with their environment via the consolidation of new positive emotional experiences. However, the mechanisms underlying interventions addressing the subjective body are far from conclusive. Again, only experimental dismantling or additive designs will illuminate changes associated with a specific type of intervention on the session level and after a treatment has ended, as already seen elsewhere (Høglend and Hagtvet, 2019). Identifying how an embodied treatment technique contributes to changes in repetitive interactional patterns could also facilitate knowledge transfer. This especially applies to clinical training, where an embodied view is often lacking, be it on the theoretical or technical side (Gennaro et al., 2019; Katzman and Coughlin, 2013).

### Strength and limitations

The quality of studies included was sufficient for all included studies, which might in part originate from the design of the review. Inclusion criteria favored studies with manualization of treatments applied in the RCTs. Designing the study around interventions on the other hand also introduced difficulties with generalizability. The only conclusion possible is one regarding an interventional repertoire (Tolin et al., 2015), which also includes interventions minding the subjective body. Regarding the meta-analytic findings, heterogeneity was a significant factor, with possible influencing factors being temporal dispersion of studies, diverse study populations and the treatment models themselves. We only accounted for the year of publication, planned number of sessions and study quality in our exploratory moderator analysis. In light of a low power resulting from the small sample size (k) of the exploratory moderator analysis (Hempel et al., 2013), these results can only point a direction for future research. Conclusions about the comparison to control conditions therefore must be drawn with caution. Another threat to the external validity of findings stems from the experimental nature of interventions studied. Given the differences between the trial and natural settings, it is reasonable to question whether interventions from the trials will be applied in the same way. For this reason, effects in other contexts may differ from those found here.

## Conclusion

Only a limited number of empirical studies have examined the efficacy of psychodynamic psychotherapies that address the subjective body in the treatment of depression. Connecting subjective experiences with the awareness of bodily states yet seems to allow for a comprehensive understanding of depressive states and the interactional misattunements associated with it. Findings from our meta-analysis suggest the potential efficacy of the respective treatments; however, conclusions can only be of a preliminary nature. Throughout the manuals used in the included trials we discerned three technical principles in approaching the subjective body but found also a vast variability in the underlying theories. Nevertheless, techniques allowing patients to move from loosely tied or sometimes weakly represented body phenomena to integrated subjective experiences need further conceptual elaboration. We suggest this task is best undertaken by developing well operationalized technical concepts that are evaluated empirically through dismantling or additive designs. Given the necessity of repetitive working through, we also belief both process and outcome studies are needed. In the meantime, the education of psychodynamic therapists could benefit from a stronger emphasis on embodied understanding.

## Data Availability

Publicly available datasets were analyzed in this study. This data can be found at: https://osf.io/dn5cq/overview?view_only=34aaf697f5fb48c5b000bd4b1eb2f465.
