# Supplementary material for: Psychodynamic treatments taking an embodied perspective and their effect on depressive symptomatology: a systematic review and meta-analysis of randomized controlled trials
Source: Front Psychol. 2026 Mar 26;17:1727473. doi: 10.3389/fpsyg.2026.1727473 (PMC13062308; doi:10.3389/fpsyg.2026.1727473)
Supplement: Supplementary file 1 [file Supplementary_file_1.docx]

**Supplemental Material**

**Search string used for databases**

PsychInfo & Medline were searched for academic journal articles written in eighter German or English, using the following Searchstring:

(psychodyn* OR dynamic OR psychoanalytic OR psychoanalysis OR analytic OR mentaliz* OR relational OR integrat*) AND (intervention OR therap* OR treatment OR psychotherapy) AND (body OR bodily OR embodi* OR affect* OR emotion* OR experiential OR experiencing OR interpersonal) AND (depress* OR dysthym*) AND (rct OR randomized OR controlled OR clinical)

**Table S1. Additional Study Characteristics**

| Author | Year | Control/BFT | Method of Analysis | Total RCT-PQRS Score |
| --- | --- | --- | --- | --- |
| Ajilchi et al. | 2016 | Control | As Treated | 32,5 |
| Barkham et al. | 1999 | BFT | As Treated | 32 |
| Driessen et al. | 2013 | BFT | ITT | 44,5 |
| Fonagy et al. | 2020 | Control | ITT | 36 |
| Gibbons et al. | 2012 | Control | ITT | 28,5 |
| Herrmann-Lingen et al. | 2016 | Control | ITT | 34,5 |
| Heshmati et al. | 2023 | Control | ITT | 38,5 |
| Meganck et al. | 2023 | BFT | ITT | 42,5 |
| Shapiro et al. | 1994 | BFT | As Treated | 34,5 |
| Town et al. | 2017/2020 | Control | ITT | 44 |
| Wang Y et al. | 2023 | Control | ITT | 36 |

Abbrevations: BFT=Bona Fide Therapy, ITT= Intention to treat, RCT-PQRS= RCT of Psychotherapy Quality Rating Scale; Additional information: the RCT-PQRS’ cut-off for a reasonably well executed Study ≥ 24 points

**Table S2: Moderators of Effects Post Treatment comparing PDP vs. Control**

| Model Parameter | estimated β | Se | Z | p | 95 % CI | |
| --- | --- | --- | --- | --- | --- | --- |
| y_i_~ Treatment Sessions intended | | | | | | |
| intercept | 0.3738 | 0.4814 | 0.7764 | 0.4375 | -0.5698 | 1.3173 |
| Sessions Intended | **-0.0620** | 0.0307 | -2.0225 | **0.0431*** | -0.1222 | -0.0019 |
| y_i_~ Year of publication | | | | | | |
| Intercept | 170.9170 | 115.0211 | 1.4860 | 0.1373 | -54.5202 | 396.3542 |
| Year of publication | -0.0849 | 0.0570 | -1.4906 | 0.1361 | -0.1966 | 0.0267 |
| y_i_~ Study Quality (PQRS) | | | | | | |
| intercept | 1.2913 | 1.8138 | 0.7119 | 0.4765 | -2.2636 | 4.8462 |
| PQRS | -0.0511 | 0.0503 | -1.0152 | 0.3100 | -0.1497 | 0.0475 |

* indicates p < .05
